# Urinary function and quality of life after radiotherapy for prostate cancer in patients with prior history of surgical treatment for benign prostatic hyperplasia

**DOI:** 10.1186/s13014-018-1149-0

**Published:** 2018-10-24

**Authors:** Mélanie Guilhen, Christophe Hennequin, Idir Ouzaid, Ingrid Fumagalli, Valentine Martin, Sophie Guillerm, Pierre Mongiat-Artus, Vincent Ravery, François Desgrandchamps, Laurent Quéro

**Affiliations:** Radiation Oncology Department, Saint Louis Hospital, 1, avenue Claude Vellefaux, 75010 Paris, France

**Keywords:** Radiotherapy, TURP, Prostate, Cancer, IPSS, Toxicity, Quality of life, Hypertension, Dose

## Abstract

**Background:**

To evaluate long-term IPSS score and urinary quality of life after radiotherapy for prostate cancer, in patients with prior history of surgical treatment for benign prostatic hyperplasia (BPH).

**Methods:**

In this retrospective study, we reviewed medical records of patients treated in our department, between 2007 and 2013 with surgery for BPH followed by radiotherapy for localized prostate cancer. Patients were contacted to fill in IPSS questionnaire and they were also asked for urinary quality of life. Predictive factors known to be associated with bad urinary function were also analysed.

**Results:**

Fifty-nine patients were included in our study. Median age was 70 years. Median follow-up was 4.6 years. Median radiotherapy dose was 78 Gy (5 × 2 Gy/week). Thirty patients (48.5%) received hormone therapy in combination with RT. Main surgery indications were urinary symptoms (65%) and urinary retention (20%). Five-year biochemical-disease free survival was 75% and 5-year clinical relapse free survival was 84%. At the time of the study, the IPSS after radiotherapy was as follows: 0–7: 77.6%; 8–19:20.7%; 20–35: 1.7%. Urinary quality of life was satisfactory for 74.2% of patients. After multivariate analysis, a high dose of RT and a medical history of hypertension were associated with a poorer quality of urinary life (*p* = 0.04).

**Conclusion:**

External radiotherapy remains an appropriate treatment option without a major risk for deterioration in urinary function in patient with antecedent surgery for BPH. High dose of RT and a medical history of hypertension were associated with a poorer quality of urinary life.

## Background

External beam radiotherapy (RT) is a well-established treatment for clinically localized prostate cancer. Benign prostatic hyperplasia (BPH) is a very common urological problem in elderly patients, and transurethral resection of the prostate (TURP) is the most common type of surgery for the treatment of BPH. Thus, many patients may have a personal medical history of TURP or significant urinary symptoms at the time of radiotherapy for prostate cancer treatment.

Currently, TURP still represents the gold standard in the operative management of BPH. The major two late complications of TURP are urethral strictures (2.2–9.8%) and bladder neck contractures (0.3–9.2%) [1]. However, it has been reported up to 29% of urethral stricture in patients who had radiotherapy after TURP [2]. Mechanical perturbations of the mucosa leading to scarring remain the most commonly reported causative factor, and fibroblast proliferation suggests that a history of multiple TURPs would be a significant risk factor for higher urinary toxicity after RT.

TURP after radiotherapy is commonly associated with poor urinary function outcome. Liu et al. reported patients treated by TURP after RT, may have 5 times more long-term urinary incontinence [3]. Several studies observed an increase urinary morbidity incidence in case of previous medical history of TURP before RT but authors did not conclude to a major risk [4, 5].

Urinary quality of life evaluation is not usually reported in studies combining TURP and radiotherapy. In the present study, we report on International prostate symptom score (IPSS) and long-term urinary quality of life of patients treated with high dose 3D RT with prior medical history of TURP.

## Methods

### Study setting

We retrospectively identified from our medical database patients treated with radiotherapy between November 2008 and February 2013, for non-metastatic prostate cancer. To be included, patients had to have biopsy proven prostate cancer and a prior medical history of TURP or adenectomy within 4 years before the diagnosis of prostate cancer. Patients treated with androgen deprivation therapy (ADT) before RT start could be included but patients with previous history of prostatectomy or prostate brachytherapy were excluded from the study.

### Treatment and assessment

The primary endpoint of our study was late urinary function evaluation (> 2 years after RT) using IPSS. The IPSS is based on the answers to 7 questions concerning urinary symptoms (score 0 to 35) Urinary symptoms of patients can be classified as follows: mildly symptomatic (IPSS score = 0–7) moderately symptomatic (IPSS score = 8–19) or severely symptomatic (IPSS score = 20–35). We evaluated the urinary quality of life question using the eighth question of the IPSS (QOL IPSS). QOL IPSS is rated from 0 (Delighted) to 6 (Terrible). Long-term urinary toxicity (urinary tract pain, macroscopic hematuria, urinary incontinence and urinary retention), using Common Terminology Criteria for Adverse Events (CTCAE) version 4.0 grading system [6] was also evaluated. In our study, we analyzed factors correlated with poor late urinary function defined by both an IPSS ≥7 and QOL IPSS ≥3. Following factors were analyzed: initial prostate volume, volume of the prostate removed by TURP, time interval between surgical procedure and radiotherapy, radiotherapy dose to the prostate, volume of the clinical target volume (CTV) (in cubic centimeter), volume of the bladder receiving 70 Gy (B70Gy), and comorbidities (Diabetes mellitus and arterial hypertension).

All patients underwent a planning computed tomography scan before RT and were treated by 3D RT to a median dose of 78.5 Gy (range, 70 to 80 Gy), prescribed to the planning target volume (PTV), according to ICRU 50 and 62 reports. Patients received initial treatment to the prostate and seminal vesicles alone (40–46 Gy) followed by a prostatic boost. A PTV expansion was typically 1 cm in all directions around the clinical target volume, except 0.5 cm posteriorly. Patients who had a high risk for regional lymphatic involvement (ie ≥15%) according to Roach formula [7], also received 44–46 Gy to the pelvic lymph-nodes. RT treatment was delivered with six individually shaped coplanar fields, with 18 MV X-rays in daily fractions of 2 Gy, 5 days per week. To minimize toxicity, dose–volume histograms were used to evaluate the dose to the rectum, the bladder, the femoral heads and the bowel. Prostate localization during RT treatment was done once weekly by portal imaging or Cone beam computerized tomography (CBCT) depending on the time period during which the patients were treated. Acute urinary toxicity was scored according to Common Terminology Criteria for Adverse Events (CTCAE) version 4.0.

The use of androgen deprivation therapy (ADT) was at the discretion of the physician.

### Statistical analysis

Statistical analyses were performed using the Stata software version 14.1 (stataCorp, TX, USA). Univariate analysis was performed using Student t-test for the quantitative variables and the Chi-2 test for the qualitative variables.

Bravais-Pearson correlation coefficient was used to confirmation a correlation between variables (correlation coefficient (r)). Statistical significance was set at a *p*-value less than 0.05.

## Results

**Fig. 1:**
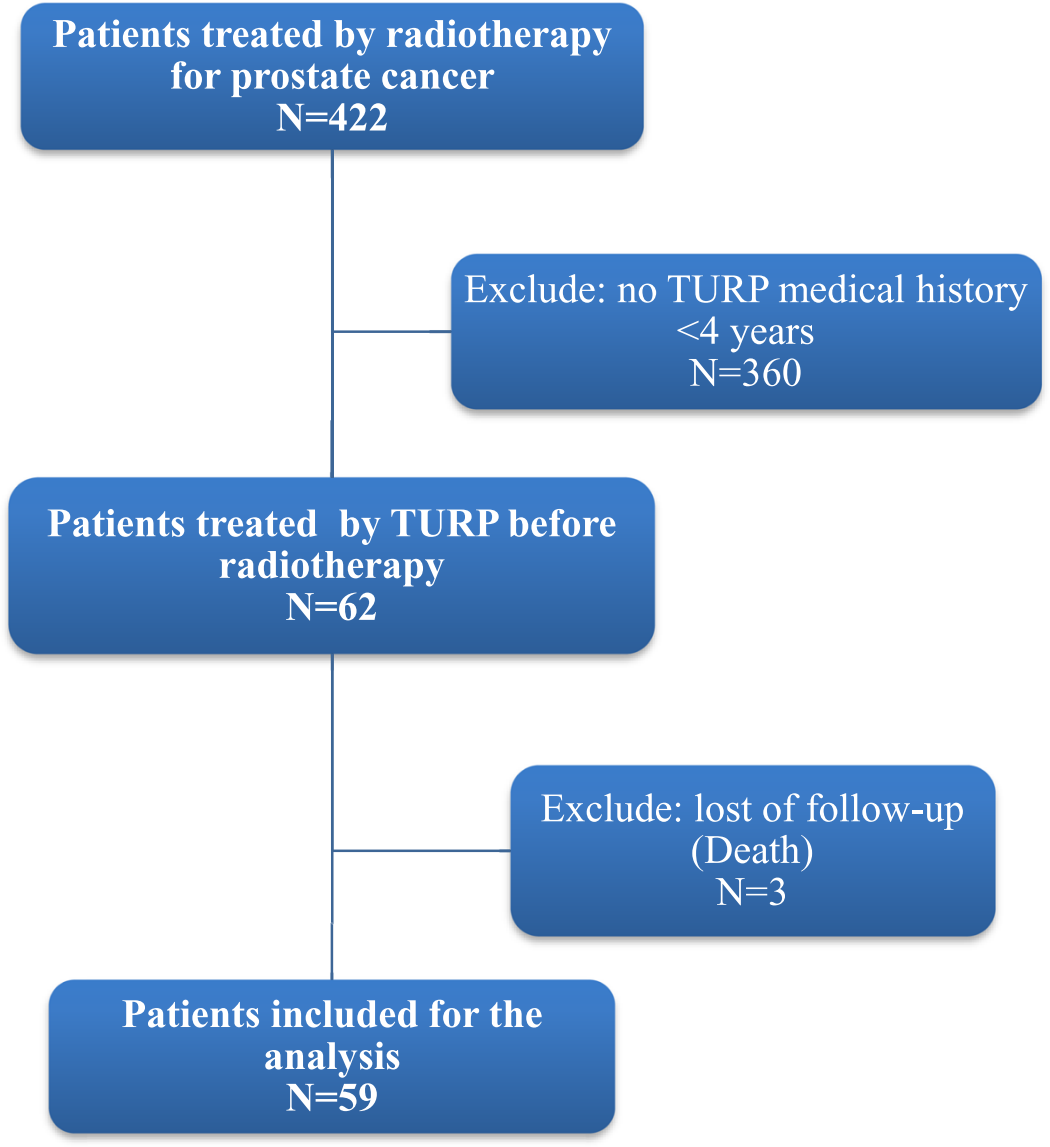
Flow chart of patient selection, with exclusion criteria

During the study period, 422 patients treated by radiotherapy in our department for non-metastatic prostate cancer were identified. Sixty-two patients who had a prior history of prostate surgery for BPH (TURP or adenectomy) were included for analysis. The indication for prostate surgery was urinary disorders in 38 patients, acute urinary retention in 11 patients, a large prostate in 4 patients and unknown reason in 11 patients. Median follow-up of the study was 4.6 years after the end of RT (range 2.2–6.9 years). Median time interval between surgery and RT was 10.6 months (range 2.2–51.6). The characteristics of patients are listed in Table 1. Thirty-three patients had arterial hypertension and 19 were treated by antihypertensive drugs. Among patients treated for hypertension, 12, 6, 9 and 5 patients were treated by angiotensin receptor blockers, beta-blockers, calcium channel blockers and diuretics respectively. Only one patient was treated by alpha blockers. Some patients received combination of multiple classes of antihypertensive drugs. Five-year biochemical disease-free survival was 75% and 5-year clinical relapse free survival was 84%. During the follow-up, 10 patients had biochemical recurrence, 7 patients had loco-regional progression, 3 patients had metastatic progression and 3 patients died: one from cardio-vascular cause and two from unknown causes unrelated to cancer. Patients who have died during the follow-up were excluded from the functional analysis (Fig. 1). The incidence of acute urinary toxicity was 45% (26/58), 45% (26/58), 8.5% (5/58), 1.5% (1/58) for grade 0, 1, 2 and 3 respectively. There was no acute grade 4 urinary toxicity.

**Table 1:** Patient characteristics

Median age (years)	69,9 [58.6–78.3]
D’Amico risk stratification	
Low risk	13 (21%)
Intermediate risk	23 (37%)
High risk	26 (42%)
Median pre-treatment PSA level	13.7 ng/mL [1.5–94]
Gleason score	
6	22 (35.5%)
7 (3 + 4)	18 (29%)
7 (4 + 3)	9 (14.5%)
≥ 8	13 (21%)
T Stage	
T1a/b	5 (8%)
T1c	27 (43.5%)
T2	20 (32.5%)
T3a	5 (8%)
T3b	5 (8%)
N Stage	
N0	60 (96.7%)
N1	2 (3.3%)
Prostate surgery	
TURP	52 (84%)
Vaporization	2 (3%)
Adenectomy	8 (13%)
Androgen deprivation therapy	30 (48.5%)
Diabete mellitus (*n* = 61)	11 (18%)
Arterial hypertension (n = 61)	33 (54%)
Medical treatment^a^	7 (11.3%)
Prostatic volume before surgery (*n* = 59)	59.5 cc [3–150]
Prostatic volume after surgery (*n* = 54)	26.8 cc [3–100]
Median RT dose	78.5 Gy [70–80]
70 Gy	3 (4.9%)
76 Gy	15 (24.2%)
80 Gy	44 (70.9%)
Whole-pelvis RT	6 (9.6%)
Bladder volume receiving 70 Gy (*n* = 57)	34.7 cc [1–86]

*RT* Radiotherapy, TURP (Trans-urethral resection of prostate)

^a^ alpha blocker or anticholinergic (solifenacin succinate)

**Fig. 2:**
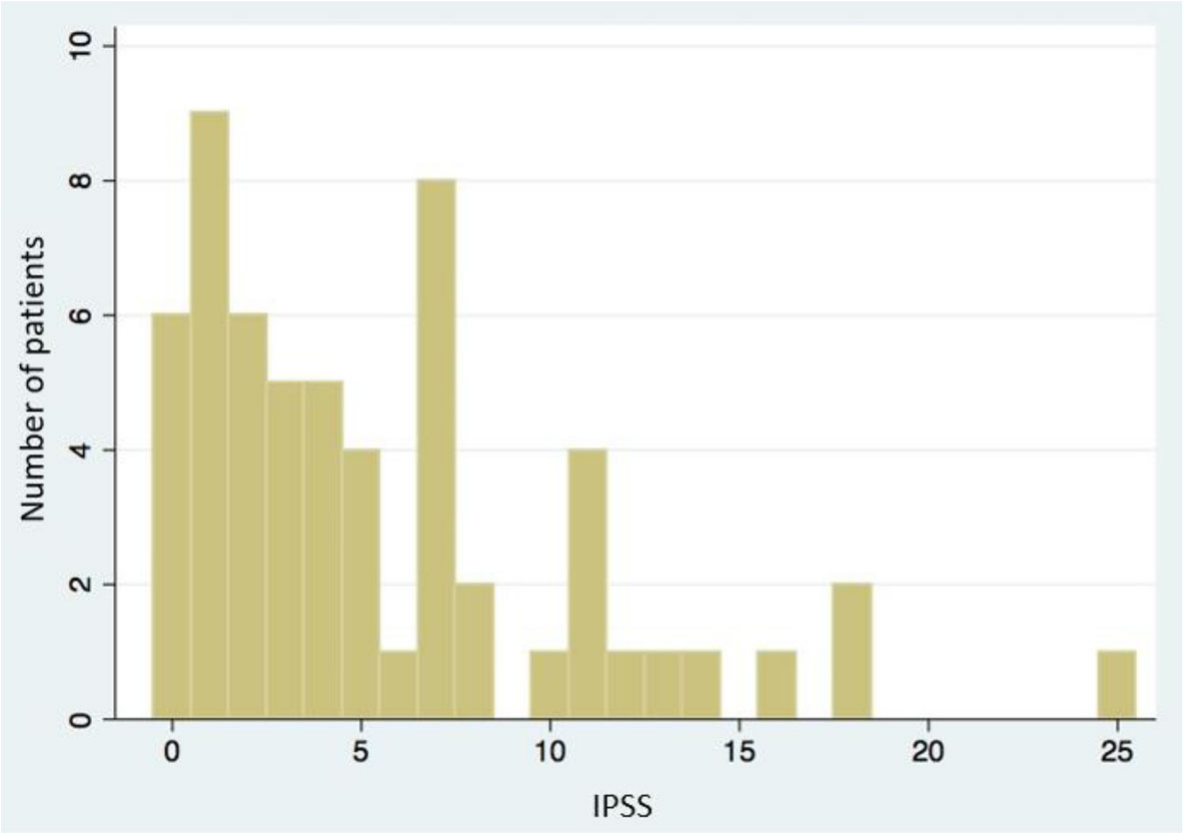
IPSS among patient population

**Fig. 3:**
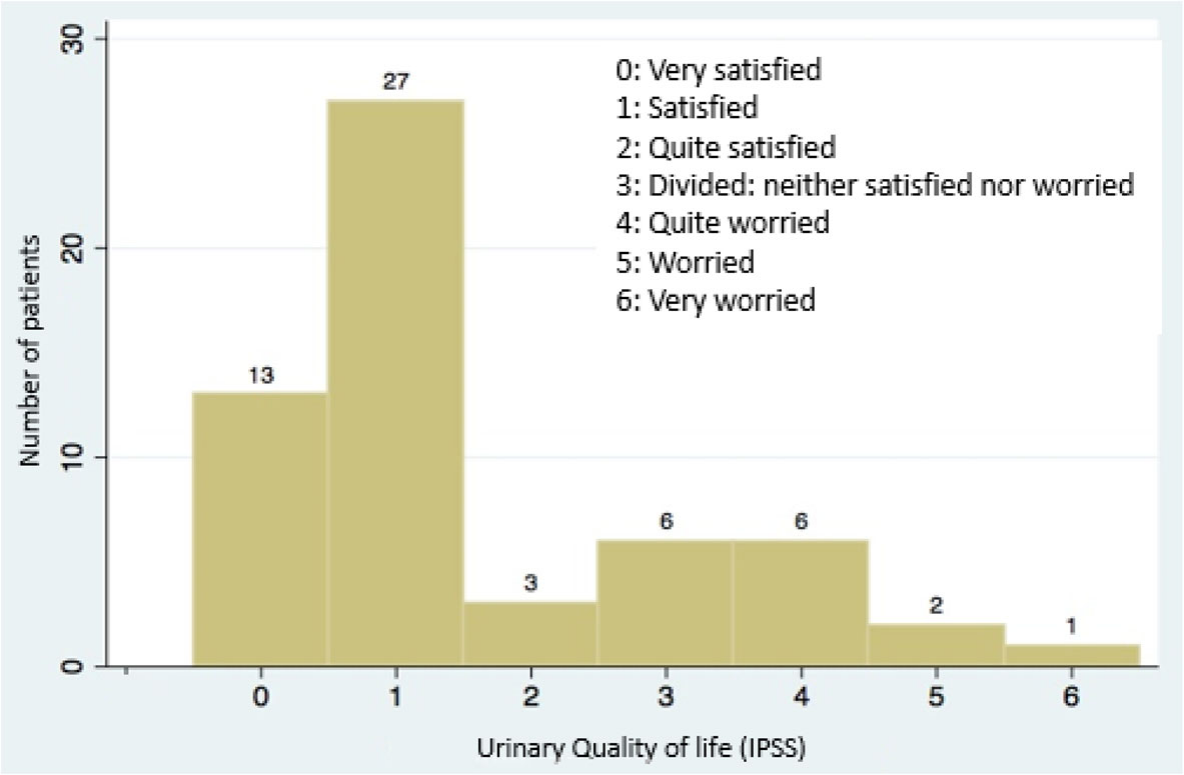
Urinary Quality of Life among patient population

**Fig. 4:**
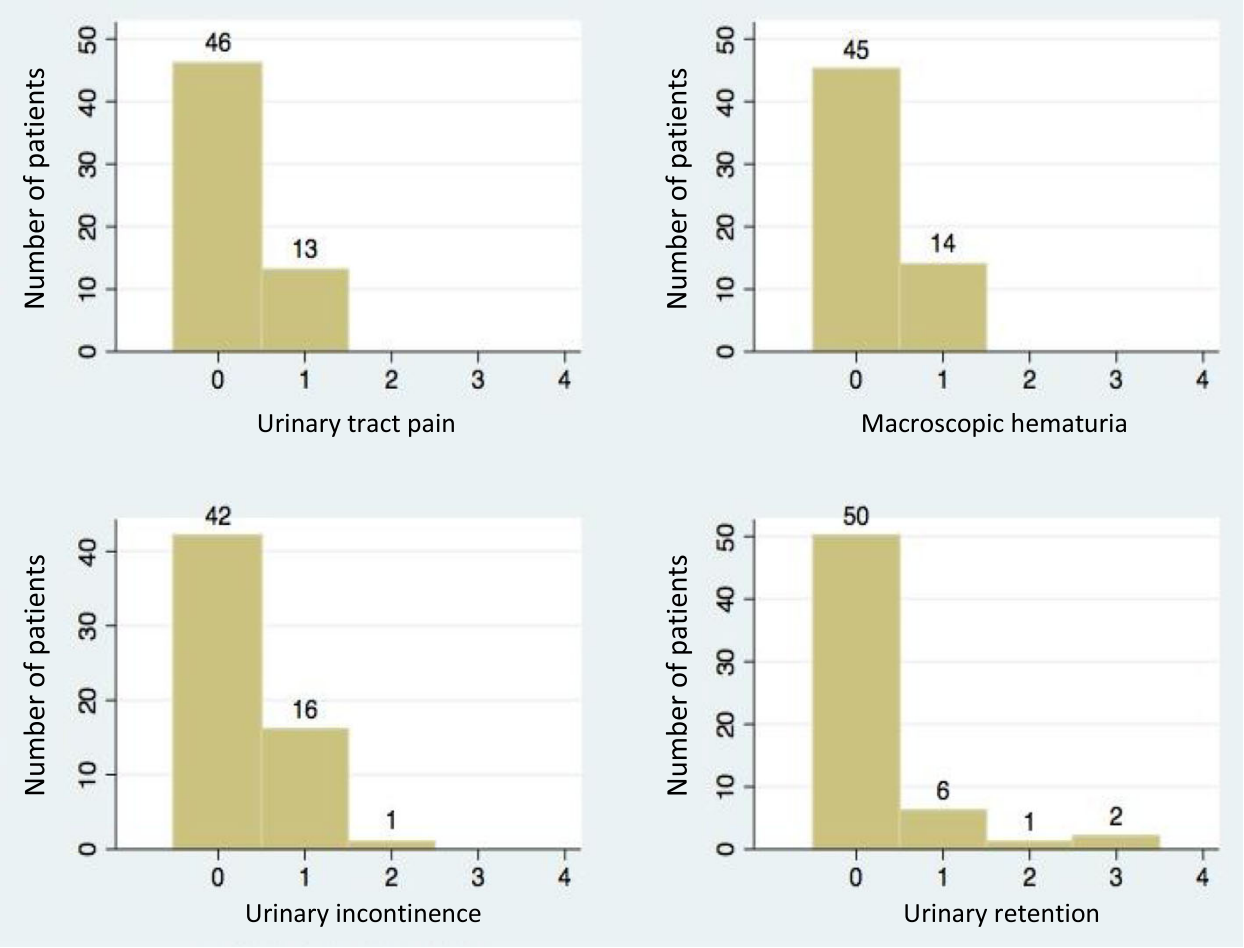
Urinary toxicity according to the CTCAE v4 grading system

Fifty-nine living patients were evaluable for QoL IPSS and long-term urinary toxicity. Among those patients, ten had biochemical relapse. One patient could not answer to the IPSS questionnaire because he had a urinary sheath*.* Median time interval between the end of radiotherapy and the urinary function evaluation by questionnaire was 4.5 years +/− 1.1. The median IPSS was 5.5 (range 0–25): 45 patients had mild urinary symptoms 45/58 (77.6%), 12 had moderate urinary symptoms (12/58 (20.7%)) and 1 had severe urinary symptoms (1/58 (1.7%)) (Fig. 2). Quality of urinary life according to the eighth question of the IPSS was as follows: Forty-three patients reported to have a good urinary quality of life (QOL IPSS < 3) (3/58 (74.1%)) but 15 patients reported to have a poor urinary quality of life (QOL IPSS ≥3) ((15/58) (25.9%)) (Fig. 3). A statistically significant correlation was observed between the IPSS score and the QOL IPSS (*r* = 0,56; *p* = 0.00001). Fifty-five patients (55/59 (93.2%)) had grade < 2 long-term urinary toxicity according to the CTCAE scale and only 4 patients ((4/59 (6.8%)) had grade ≥ 2 long-term urinary toxicity (Fig. 4). One patient and three patients had grade 2 and grade 3 urinary retention respectively and one patient had grade 2 urinary incontinence. No patient had grade 4 long-term urinary toxicity.

**Fig. 5:**
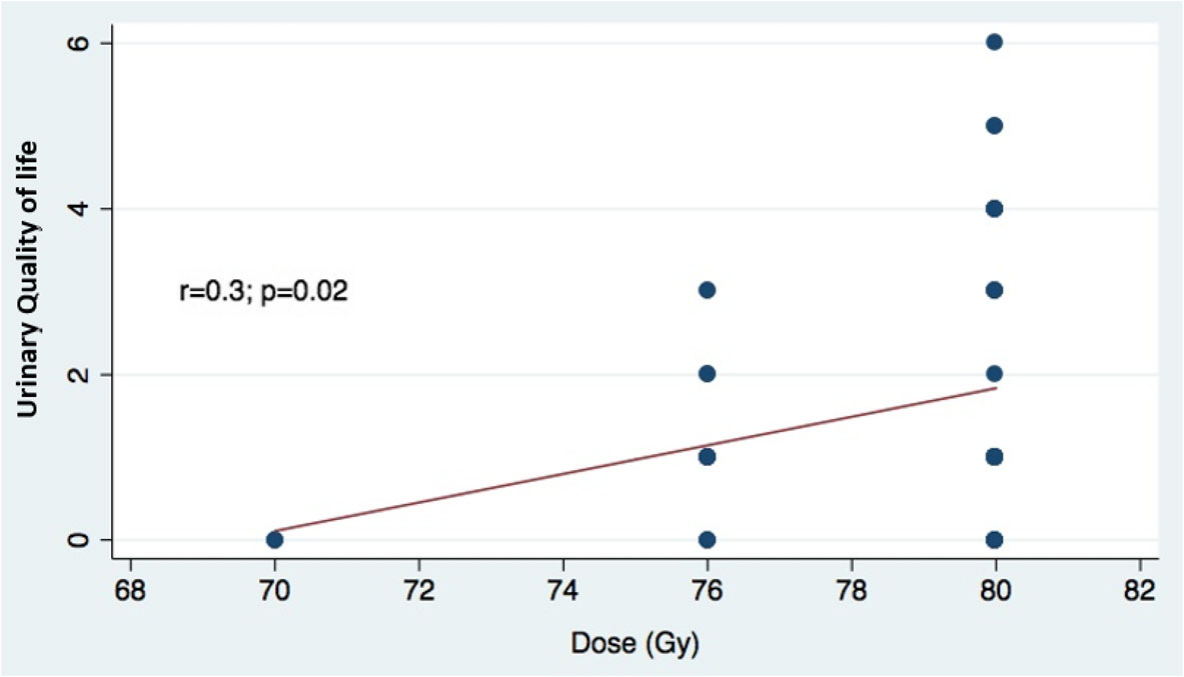
Urinary Quality of life and radiotherapy dose

A significant correlation was observed between RT dose and the QOL IPSS (*r* = 0.3; *p* = 0.02) (Fig. 5) but not between RT dose and the IPSS.

A significant correlation was also observed between the arterial hypertension (HTA) and the QOL IPSS (*p* = 0.04) (Table 2) but not between the acute urinary toxicity and the QOL IPSS (*p* = 0.064). There was no correlation observed between diuretic intake and QOL IPSS (*p* = 0.167). Three patients with HTA were excluded from the analysis because they have died during the follow-up. We also found a correlation between HTA and IPSS> 7 but this correlation was not statistically significant (*p* = 0.5) (Table 2). It is important to note that the nine patients who had medical history of HTA and a QOL IPSS≥3 did not take diuretics at the time of RT.

**Table 2:** Risk factors associated with long term urinary toxicity (Univariate analysis)

	IPSS ≤7	IPSS > 7	p	QOL < 3	QOL ≥3	p	CTCAE Grade < 2	CTCAE Grade ≥ 2	p
Initial prostate volume (g)	56	71	0.06	58	64	0.24	60	36	0.08
Resected prostate Volume (g)	28	24	0.3	26	30	0.2	27	16	0.1
CTV prostate (cc)	45	61	0.07	50	59	0.1	53	37	0.08
V70Gy (cc)	34	37	0.2	35	36	0.4	34	40	0.2
TURP-RT time interval (days)	312	398	0.2	312	382	0.2	306	614	0.05
Total dose of radiotherapy (Gy)	78.3	78.7	0,3	78	79.4	0.04	78.4	79	0.3
Hypertension									
No	24	3	0.5	21	6	0.04	27	0	0.05
Yes	20	10		21	9		27	4	
TURP indication									
Unknown	5	0	0.1	4	1	0.1	5	0	0.8
Urinary symptoms	28	10		26	12		36	3	
Urinary retention	10	1		11	0		10	1	
High prostate volume	2	2		2	2		4	0	
Androgen deprivation therapy									
No	23	9	0.2	25	6	0.2	29	3	0.3
Yes	22	4		18	9		26	1	

*IPSS* International prostate symptom score, *QOL* urinary quality of life, *CTCAE* (Common Terminology Criteria for Adverse Events) version 4 grading system, *TURP* transurethral resection of the prostate

A non-significant trend was found between an IPSS < 7 and a small prostate volume (p = 0.06). We found no significant correlation between a poor lower urinary function and the prostate volume after surgery for BPH, the B70Gy, the prostate CTV, the time interval between surgery and RT, diabetes mellitus and androgen deprivation therapy.

## Discussion

In our study, we reported that almost 75% of patients had a good urinary quality of life after RT (QOL IPSS < 3), despite a previous medical history (PMH) of surgery for BPH.

In the literature, the results of the studies about toxicity after RT in patients previously treated by TURP are debated: the late urinary incontinence rate varies from 0 to 13.3% (Table 3).

**Table 3:** Studies on toxicity after radiotherapy in patients previously treated by TURP

Authors	Years	n	Dose RT	Late urinary incontinence rate
Gibbons et al. [24]	1979	71	66.8 Gy (Mean)	0%
Perez et al. [9]	1980	60	60–70 Gy	13.3%
Pilepich et al. [25]	1981	88	65–70 Gy	2.3%
Green et al. [26]	1990	130	65 Gy	5.5%
Amdur et al. [27]	1990	114	65–70 Gy	3%
Perez et al. [8]	1994	242	62–72%	2%
Lee et al. [5]	1996	132	68–79 Gy	2%
Sandhu et al. [11]	2000	120	64.8–81 Gy	10%
Liu et al. [3]	2005	246	66 Gy (median)	10%
Devissety et al. [2]	2010	71	70 Gy (Median)	3%
Current study	2018	59	78 Gy (Median)	2%

Lee et al. reported on a late urinary incontinence rate of 2% vs 0.2% after RT in patients with or without a PMH of TURP respectively. Despite a lower incidence of late urinary incontinence rate, the difference was significant [5]. In two independent studies, Perez et al. reported on a non-significant increase in the incontinence rate: 13% vs 4% in the first study and 2% vs 0% in the second study [8, 9]. It is important to note that all patients in these studies were treated with old 2DRT technics. In a study on 1192 patients, published in 2004, Liu et al. evaluated urinary incontinence in patients treated by 2DRT or 3DRT for prostate cancer. Two hundred and forty-six patients had a previous medical history of TURP. Grade ≥ 1 incontinence rate at 5 years was greater in patients with a PMH of TURP in comparison with those without PMH of TURP (10% vs 6%, *p* = 0.03) [3]. N. et al. evaluated 285 patients treated with 3DRT at a dose of 79.2Gy, of whom, of whom 13% had a TURP beforehand [10]. Grade 2–3 urinary toxicity at 5 years was greater for patients with PMH of TURP (25.7% vs 6.1%, *p* = 0.0002).

S. et al., did not find any difference regarding grade ≥ 2 long-term overall urinary toxicity in patients treated by 3DRT or IMRT with or without a PMH of TURP (10 vs 9%) [11].

In a systematic review of the literature, 13 studies among 14, reported a higher incontinence rate in patients with PMH of TURP compared with patients without PMH of TURP. Among these studies, four demonstrated a statistically significant increase of incontinence [4]. Acute grade ≥ 2 urinary toxicity, longer follow-up time, and stage ≥T3 were reported as risk factors for incontinence in this group. In our study, high RT dose to the prostate was associated with a long-term poor urinary QOL. Indeed, in our study, patients with an IPSS> 7 received a higher average dose to the prostate and the 3 patients who received only 70 Gy to the prostate had a QOL IPSS = 0. Despite a relatively high median dose delivered to the prostate (78.6 Gy) with 70.9% of patients receiving 80 Gy and 24.2% 76 Gy, most of the patients were overall satisfied with their urinary QOL.

D. et al. reported on a study of 609 patients, treated by RT for prostate cancer, a non-significant increase risk of urinary toxicity in patients with a PMH of TURP or a RT dose greater than 74 Gy [2]. In several studies it was shown that the RT dose is associated with a higher urinary toxicity. Z. et al. reported on a higher urinary toxicity grade ≥ 2 rate for patients treated with a dose > 75.6 Gy delivered by 3DRT (14% vs 5%, *p* < 0.001) [12, 13].

However, modern irradiation technics such as Intensity Modulated Radiotherapy (IMRT)/ Volumetric Modulated Arc Therapy (VMAT)/ Image Guided Radiotherapy (IGRT) could decrease late urinary toxicity rate in patients treated with high-dose radiotherapy. Zapatero et al. reported that compared with 3DCRT, high-dose IMRT/IGRT was associated with a lower rate of late urinary complications (6.4% vs 10.8%, *p* = 0.056) in spite of higher radiation dose (80.7 Gy vs 78.7 Gy, p < 0.001) [14]. In patients treated with high dose IMRT (86.4 Gy) + IGRT, Z. et al. have reported a lower rate of 3-year likelihood of grade 2+ urinary toxicity in comparison with patients treated without IGRT (10.4% vs 20.0%, *p* = 0.02) [15].

Few studies have both evaluated urinary function and quality of life using the IPSS questionnaire after definitive RT for prostate cancer. In our study, we also evaluated QOL IPSS, to take into account the patient’s point of view about their urinary toxicity. In our study, 45/58 (77.6%) of patients had a mild, 12/58 (20.1%) a moderate and 1/58 (1.7%) a severe IPSS. For the urinary QOL IPSS, 43/58 (74%) were satisfied, 6/58 (11%) were neither satisfied nor worried and 9/58 (15%) were quite worried to very worried.

Despite the PMH of TURP, only one patient had a severe IPSS, the vast majority of our patients had a mild IPSS and a good urinary quality of life. Our results are comparable with those published by L. et al., who reported IPSS in 154 patients treated by 3DRT or 2DRT without TURP: 79% of patients had a mild IPSS score, 14% had a moderate IPSS score and 6% had a severe IPSS score. For the urinary QOL IPSS, 87% of the patients were satisfied, 10% were shared and 3% were annoyed [16]. In a study of 60 patients treated by 3DRT without TURP for prostate cancer, Pastorello et al. reported at 48 months after RT, a median IPSS = 11. The median IPSS of their patients was higher compared with the median IPPS of our patients treated by TURP and RT (11 vs 5.5) [17].

We also found in our study, a correlation between a worse QOL IPSS and a PMH of hypertension. Our results are in accordance with Cozzarini et al., who observed in a study of 742 patients more grade 2–3 late urinary toxicity in patients with hypertension and treated with postoperative RT [18]. Hypertension has also been found to be associated with an increased risk of urethral stricture after prostate brachytherapy [19].

It has been observed that circulating catecholamine levels are high in patients with arterial hypertension [20, 21]. Increase catecholamine levels may influence the lumbosacral cord and therefore increase urinary frequency [22].

No other statistically significant risk factors for long-term urinary toxicity were found.

Due to the retrospective nature of our study, baseline IPSS before RT was not available, therefore we did not evaluate the relationship between baseline IPSS and post RT IPSS or QOL IPSS.

Chevli et al. who evaluated 368 patients treated with RT, did not show an association between the volume of the prostate gland (VP) before treatment and urinary toxicity at 1 year [23]. VP does not appear to be a good indicator of radiotherapy toxicity in patients with or without TURP.

The absence of correlation between surgery-RT delay and long-term urinary function observed in our study could be explained by our local procedure, which recommends a minimum delay between the TURP and the start of radiotherapy of 10 or more weeks, to allow sufficient time for the tissues to heal.

Our study has a number of limitations, including its retrospective nature, a small sample and the fact that it was conducted at a single institution. Patients have been questioned on their urinary function at different interval after RT.

## Conclusion

RT in patients previously treated by surgery for BPH is feasible, well tolerated and is associated with low IPSS score, a satisfactory quality of life and a low incidence of severe long-term urinary toxicities, similar to those without surgery for BPH, reported in the literature. External radiotherapy remains an appropriate treatment option without a major risk for deterioration in urinary function in patient with antecedent surgery for BPH and with prior medical history of arterial hypertension. In practice, for patients with poor urinary function, surgery may be a good option to alleviate urinary symptoms, albeit requiring a waiting time to heal before RT.

## Abbreviations

ADT: Androgen deprivation therapy
BPH: Benign prostatic hyperplasia
CBCT: Cone beam computerized tomography
CTCAE: Common terminology criteria for adverse events
CTV: Clinical target volume
ICRU: International commission on radiation units and measurements
IPSS: International prostate symptom score
PMH: Previous medical history
PTV: Planning target volume
RT: External beam radiotherapy
TURP: TransUrethral Resection of the Prostate
